# A Syringe-Based Biosensor to Rapidly Detect Low Levels of *Escherichia coli* (ECOR13) in Drinking Water Using Engineered Bacteriophages

**DOI:** 10.3390/s20071953

**Published:** 2020-03-31

**Authors:** Troy C. Hinkley, Spencer Garing, Paras Jain, John Williford, Anne-Laure M. Le Ny, Kevin P. Nichols, Joseph E. Peters, Joey N. Talbert, Sam R. Nugen

**Affiliations:** 1 Department of Food Science, Cornell University, Ithaca, NY 14853, USA; thinkley@intven.com; 2 Intellectual Ventures Laboratory/Global Good, Bellevue, WA 98007, USA; sgaring@intven.com (S.G.); pjain@intven.com (P.J.); jwilliford@intven.com (J.W.); aleny@intven.com (A.-L.M.L.N.); knichols@intven.com (K.P.N.); 3 Department of Microbiology, Cornell University, Ithaca, NY 14853, USA; joe.pters@intven.com; 4 Department of Food Science and Human Nutrition, Iowa State University, Ames, IA 50011, USA; jotalber@iasta.edu

**Keywords:** bacteriophage, drinking water, E.coli, rapid detection

## Abstract

A sanitized drinking water supply is an unconditional requirement for public health and the overall prosperity of humanity. Potential microbial and chemical contaminants of drinking water have been identified by a joint effort between the World Health Organization (WHO) and the United Nations Children’s Fund (UNICEF), who together establish guidelines that define, in part, that the presence of *Escherichia coli* (*E. coli*) in drinking water is an indication of inadequate sanitation and a significant health risk. As *E. coli* is a nearly ubiquitous resident of mammalian gastrointestinal tracts, no detectable counts in 100 mL of drinking water is the standard used worldwide as an indicator of sanitation. The currently accepted EPA method relies on filtration, followed by growth on selective media, and requires 24–48 h from sample to results. In response, we developed a rapid bacteriophage-based detection assay with detection limit capabilities comparable to traditional methods in less than a quarter of the time. We coupled membrane filtration with selective enrichment using genetically engineered bacteriophages to identify less than 20 colony forming units (CFU) *E. coli* in 100 mL drinking water within 5 h. The combination of membrane filtration with phage infection produced a novel assay that demonstrated a rapid, selective, and sensitive detection of an indicator organism in large volumes of drinking water as recommended by the leading world regulatory authorities.

## 1. Introduction

The United Nations has determined that every person has a fundamental human right to water and sanitation [1]. Unfortunately, millions still lack access to potable water sources within reasonable walking distances from their homes [2].Not only does microbial contamination of drinking water contribute significantly to morbidity and mortality worldwide, it disproportionally affects low income countries [3].Improper sanitation of drinking water sources is strongly correlated with the presence of coliforms (a widely variable group of gram-negative rod shaped bacteria that possess a range of biochemical attributes [4]). *Escherichia coli* (*E. coli*), which is a coliform, is also a near ubiquitous resident of mammalian gastrointestinal tracts [5], and thus has been determined to be an appropriate indicator of fecal pollution from warm-blooded animals. In 2015, almost 850 million people still lacked access to a basic drinking water source [6] of those 850 million people, children typically bear the brunt of the disease burden, as their developing immune systems are dramatically less effective at eradicating ingested coliforms in improperly sanitized drinking water [7]. The resulting diarrheal disease typically results in severe dehydration, a condition that necessitates a clean drinking water source for improvement in well-being. In addition, various strains of *E. coli* are responsible for nearly a third of neonatal sepsis cases [8] and the majority of urinary tract infections worldwide [9].

Even though the detection of any indicator species will have inherent limitations and biases [10], the detection of *E. coli* as an indication of poor sanitation has been widely successful in the improvement of water supplies worldwide [2,11,12,13,14]. While standard culture-based techniques are reliable, results require anywhere from 24 to 72 h [15,16], a relative eternity for a community water supply in need of remediation [17].

The rapid detection of bacteria remains a significant challenge, and many research approaches have been developed to help address this important public health issue [18]. One promising area of rapid microbial detection assays are bacteriophage-based diagnostics [19]. Bacteriophages are obligate bacteria-infecting viruses that have co-evolved with bacteria for most (if not all) of the more 3 billion years that bacteria have existed on the planet [20]. Reporter phages have been modified to include an exogenous reporter gene in their genome. The newly created recombinant phage expresses the exogenous cellular biomarker upon phage infection, in addition to new phage progeny [21]. Measurement of the reporter enzyme activity allows for correlation between signal output (e.g., absorbance, fluorescence, and luminescence) and estimation of the number of host organisms in the sample over a certain threshold.

The field of bioluminescence has been advancing for 70 years, to where we now find McElroy’s pioneering work [22] on the development and widespread implementation of NanoLuc luciferase [23,24,25,26,27,28,29,30,31,32,33]. Luminescent reporter phages have been developed for the sensitive detection of common foodborne pathogens such as *Escherichia coli* [24,34,35], *Listeria monocytogenes* [36], *Salmonella typhimurium* [37], *Mycobacterium tuberculosis* [38], and *Vibrio parahaemolyticus* [39], among others [40,41,42,43,44,45]. The NanoLuc reporter is uniquely suited for sensitive detection assays, as it confers a low background signal coupled with high dynamic range [33].

In addition, the orthogonality of the NanoGlo chemical substrate system [33] offers consistently low background levels in a wide range of sample conditions, a characteristic enabling low-cost implementation. An issue with previous systems was that as sensitivity increased, distinguishing signals from noise became a major challenge in the establishment of the limit of detection.

The selective nature of phages has been well documented [46], and therefore our phage-based detection assay is well suited in situations where indicators and/or pathogen detection is pertinent [47]. To create the recombinant phages used in our novel detection platform, an enzyme expression cassette was inserted into a wild type phage genome to force expression of a heterologous reporter enzyme, in addition to new phage progeny upon phage infection. The NanoLuc enzyme was selected as the reporter enzyme, as it is more than 100x more active than its luminescent counterparts [33], and this highly active reporter enzyme has already ben widely deployed in a variety of detection assays [25,26,31,32], including bacteriophage-based schemes [24,48,49]. The NanoLuc enzyme was further functionalized by genetically fusing a cellulose binding module (CBM) to the C-terminus of the NanoLuc reporter gene. The CBM selected for this work (CBM2a) has previously been fused to a dimeric reporter enzyme (alkaline phosphatase), which allows for successful immobilization on cellulose while still retaining enzymatic activity [50]. In comparison, NanoLuc is a much smaller, monomeric reporter that is frequently utilized as a genetic fusion tag.

Upon phage infection, the expression levels of the reporter enzyme have a direct effect upon the limit of detection, as fewer cells are required to produce enough reporter enzyme for the signal to rise above the detection limit. As a result, we modified the upstream regulatory regions of the reporter enzyme cassette to determine the optimal sequences that permit the detection of the lowest concentration of cells. 

Herein, we propose a rapid (5 h) bacteriophage-based approach for the sensitive detection of the indicator bacteria *Escherichia coli*, the target of many regulatory requirements [51]. The novel detection assay couples membrane filtration with bacteriophage infection to generate a luminescent signal when viable *Escherichia coli* are present. The combination of sample concentration with heterologous reporter enzyme expression upon bacteriophage infection produced a detection limit of less than 20 CFU in 100 mL of drinking water.

## 2. Materials & Methods

### 2.1. Materials & Reagents

NanoGlo (luminescent substrate) was purchased from Promega (Madison, WI, USA) and prepared immediately prior to use. Regenerated cellulose filters (diameter 13 mm, pore size 0.2 µm) were fitted within polycarbonate reusable syringe filter housings (Sartorious Stedim Biotech GmbH, Goettingen, Germany) and autoclaved prior to use in detection assays. Assembled, autoclaved filters were fitted to sterile single use syringes (100 mL, Wilburn Medical, Kernersville, NC, USA) to perform filtration. All other reagents were purchased from Sigma Aldrich (St. Louis, MO, USA).

### 2.2. Bacterial Strains & Growth Conditions

*Escherichia coli* BL21 was obtained from ATCC (Manassas, VA, USA) and *E. coli* ECOR13, a reference strain of *E. coli* isolated from a healthy human, was obtained from the Thomas. S. Whittam STEC Center (East Lansing, MI, USA). Bacterial stocks were stored at −80 °C in 25% glycerol prior to use and cultured in Lysogeny Bertani high salt (LB) broth and plated on LB agar. Overnight cultures of *E. coli* BL21 and *E. coli* ECOR13 were cultivated in Luria-Bertani medium (12–16 h, 37 °C, 250 rpm). The concentration of *E. coli* ECOR13 used in detection assays was determined by standard plate counts on LB agar (24 h, 37 °C). 

Bacteriophage T7 was propagated on *E. coli* BL21 using standard protocols. Briefly, an overnight culture of *E. coli* BL21 was subcultured in LB (200 mL, 37 °C, 250 rpm, ~2 h) and grown to the midexponential phase (OD_600_ = 0.6). Phages were added to the bacterial culture at a multiplicity of infection (MOI) of 0.1, and incubated (37 °C, 250 rpm) until lysis was observed (~2 h). Cellular debris was removed via centrifugation (3200× *g*, 10 min, 4 °C) before sterile filtration (0.22 µm). Phage particles were precipitated by overnight incubation (4 °C, 18 h) with polyethylene glycol 6000 (PEG6000, 4%) and sodium chloride (NaCl; 0.4 M), before ultracentrifugation (35,000× *g*, 120 min, 4 °C). Phages were resuspended in phosphate buffered saline (1x PBS, pH 7.4) and stored at 4 °C. Standard double agar overlay assays were used to enumerate phage samples [52]. Phage stock solutions used in detection assays were diluted to 10 [9] PFU/mL in LB, sterile filtered (0.22 µm), and stored at 4 °C.

### 2.3. DNA Isolation

DNA was prepared in accordance with standard procedures [53]. Briefly, concentrated phage stocks (5 mL, >10 [11] PFU/mL) were treated with sodium dodecyl sulfate (5 mL; 4%) at 70 °C for 20 min before cooling on ice. Potassium acetate (5 mL, 2.55 M, pH 4.8) was added, the samples were centrifuged (10 min, 10,000× *g*, 4 °C), and the supernatant was applied to an anion exchange resin (Qiagen Genomic Tip 100/G), in accordance with the manufacturer’s specifications.

### 2.4. Recombinant Phage Construction

The *E. coli* codon optimized NanoLuc-CBM reporter gene cassette was synthesized and cloned in plasmid pUC57 (Genscript USA Inc.). The synthesized cassette was excised with restriction enzymes EcoRI and HindIII n and ligated to the predigested T7 Select EcoRI/HindIII adaptor DNAs (Novagen). The ligation mix was packaged in vitro using a T7 select packing extract and transduced into 300 µL (OD_600_ 0.6) of *E. coli* BL21 followed by 30 min incubation at 37 °C. Multiple dilutions of transduced *E. coli* BL21 were mixed with LB-top agar (0.75 %) and poured over LB plates. Plates were incubated at 30 °C overnight. Plates with the largest numbers of isolated plaques were selected. Individual plaques were screened for the expression of the NanoLuc-CBM cassette by adding NanoGlo substrate (Promega, Madison WI, USA), followed by imaging for 30 s (Rebel T6, Canon, Melville NY, USA) in a dark box (LTE-13, Newport Corporation, Irvine, CA, USA). The presence of luminescence on plaque periphery indicated the successful introduction of the NanoLuc-CBM cassette in the T7 phage. One individual plaque was picked, and this was followed by two rounds of plaque purification and amplification on *E. coli* BL21 [52], and referred to as NRGp5. The insertion of the NanoLuc-CBM cassette in the T7 phage was confirmed by PCR and its integrity confirmed by Sanger sequencing.

### 2.5. Phage Characterization

The genomes from the NRGp5 phages were isolated and submitted for sequencing. Characterization of the phage infection was compared to the original T7 Select. The phage host *E. coli* ECOR13 was grown from the stationary phase in LB media (3 h, 37 °C, 250 rpm) using 24 well microplates (Greiner Bio-One North America Inc., Monroe, NC, USA). The optical density at 600 nm (OD_600_) was determined at periodic time intervals to observe the growth of the bacterial cells. Phage addition (negative control, 10^7^ PFU/mL T7 Select, or 10^7^ PFU/mL NRGp5) took place at 180 min and the optical density (OD_600_) was monitored for 300 min using a Synergy Neo2 microplate reader (Biotek Instruments, Winooski, VT, USA).

The luminescence during the phage infections was monitored for a negative control, T7 Select, and NRGp5, as well as a previously developed NRGp4 [48]. Luminescent signals were measured using the same microplate reader (Biotek Instruments, Winooski, VT, USA) in sterile 24-well suspension culture plates (Cellstar, Greiner Bio-One, Monroe, NC, USA) with a 0.1 s integration time. To approximate growth conditions in the detection assay, the host *E. coli* ECOR13 was grown from the stationary phase in LB supplemented with NanoGlo (3 h, 37 °C, 250 rpm). Phage addition took place at 180 minutes and luminescence was measured at 30 min intervals for 300 min.

### 2.6. Dose Response

The sensitivity of phage NRGp5 to different concentrations of bacteria in the growth media was evaluated. Phage NRGp5 (10^7^ PFU/mL) was added to serial dilutions of mid-exponential phase *E. coli* ECOR13 (0 to 10^5^ CFU/mL) and incubated (1.5 h, 37 °C) to allow the phage infection to proceed. Aliquots were mixed 1:1 with NanoGlo substrate and luminescence was measured on a Synergy Neo2 microplate reader (Biotek Instruments, Winooski, VT, USA). Viable bacterial concentrations were confirmed using standard plate counts.

### 2.7. Infection of Non-Viable Cells

The ability of phage NRGp5 to differentiate between viable and nonviable bacterial cells was evaluated by treating identical cultures with either alcohol (inactivation) or a biological buffer (control) before phage infection. Briefly, *E. coli* ECOR13 was harvested at the mid-exponential phase (OD_600_ = 0.5), separated into identical aliquots, and centrifuged (3000× *g*, 5 min). The cell pellet was resuspended in either ethanol (70%) or phosphate buffered saline (PBS; 1×), and incubated at room temperature for 10 min. The cells were pelleted again (3000× *g*, 5 min), resuspended in sterile autoclaved drinking water (100 mL, 20 °C), and used as analytical samples for the phage-based diagnostic assay.

### 2.8. Phage-Based Syringe Filter Detection Assay

**Figure 1:**
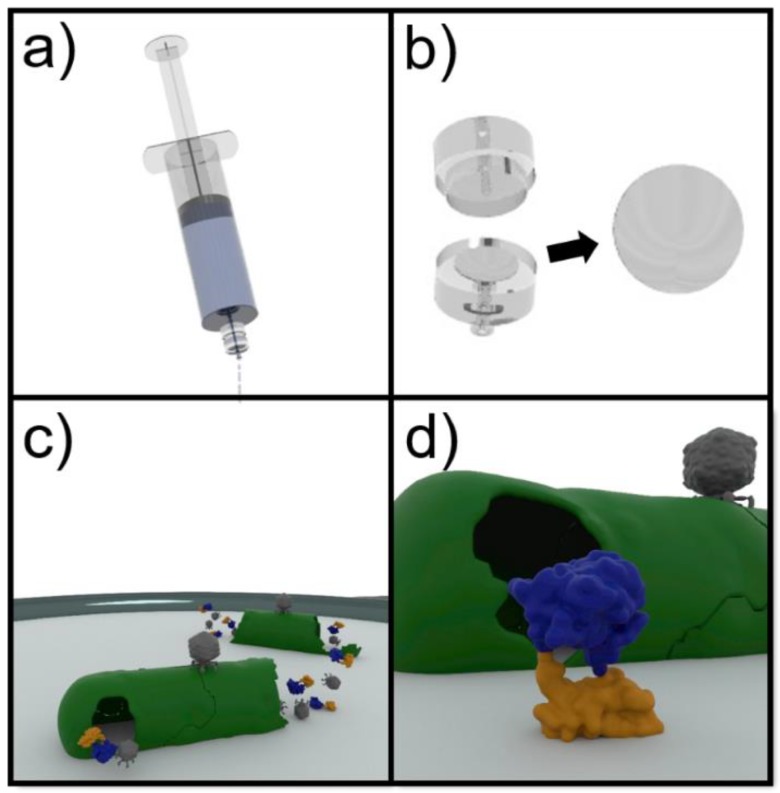
Assay format for the detection of *E. coli* in drinking water. (**a**) The water is filtered through a 0.22 µm cellulose filter in order to separate the bacteria. (**b**) The filter is then removed from the housing and placed on LB media in order to resuscitate the trapped bacteria. (**c**) Following the application of the engineered phages (grey), an infection cycle results in the expression and release of a reporter enzyme consisting of NanoLuc (blue) and a carbohydrate binding module (orange) with specificity to cellulose. (**d**) The fusion enzyme binds to the cellulose filter and the luminescent activity can then be determined.

An illustrative schematic for the detection scheme is detailed in Figure 1. Drinking water was autoclaved to account for any natural flora before aliquots (100 mL) were deliberately spiked with known concentrations of *E. coli* ECOR13. Samples were then passed through 0.22 µm regenerated cellulose filters (Sartorius Stedim Biotech GmbH, Goettingen, Germany) to separate the bacteria. The filters were removed and incubated on LB media (3 h, 37 °C) in order to resuscitate the bacteria. Phage NRGp5 (10^7^ PFU/mL) was applied to the enriched bacteria on the filter and incubated (1.5 h, 37 °C) for the expression of the luminescent reporter. The filters were fully submerged in NanoGlo substrate and luminescence was measured every 12 s for 5 min in a spectrophotometer to capture peak signal generation. The variability of the blank was used to calculate the limit of detection using the standard method of adding three times the standard deviation of the blank to the mean blank value.

## 3. Results & Discussion

### 3.1. Recombinant Phage Construction

NanoLuc, was selected as a preferred reporter because it is a small, highly active, monomeric enzyme [33]. The 19 kDa luciferase was genetically fused to a carbohydrate binding module to create a 31 kDa fusion enzyme. This novel fusion reporter has demonstrated the simultaneous capability to generate a luminescent signal while specifically bound to a cellulosic substrate. The whole phage genome sequencing results of reporter phage NRGp5 revealed no mutations to the insertion cassette, as well as no insertions, deletions, or significant mutations outside of the cloning site.

### 3.2. Phage Characterization

**Figure 2:**
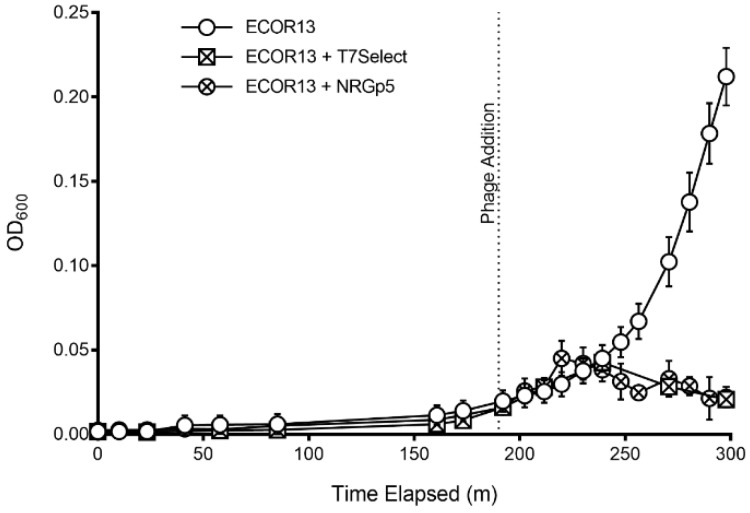
Phages (NRGp5 or T7 Select) were added to individual cultures of *E. coli* (ECOR13) after 3 h of enrichment. The *E. coli* in samples without phages added continued to the exponential phase, while the application of phages resulted in a decline in optical density after about two hours.

Initial screening for the successful insertion of the reporter gene was determined by visualizing the luminescence of phage plaques. Reporter gene expression was confirmed via long exposure photography of double overlay plaque assays where NanoGlo substrate was directly applied to the isolated plaques. Luminescent plaques were isolated and propagated to create high-titer stocks for downstream use, including detection assays and DNA isolation for whole genome sequencing. The ability of phage NRGp5 to infect and lyse indicator bacteria was directly compared to the original T7 Select, to evaluate if the addition of the reporter gene had a measurable effect on the apparent fitness of the phage. As seen in Figure 2, the lysis profiles of the phages are similar, indicating that the reporter gene insertion had a minimal effect on the ability of the phage to lyse infected bacteria. Furthermore, the burst size of phage NRGp5 was similar to that of NRGp4 and T7 Select (data not shown). The genetic location of the NanoLuc-CBM gene insertion was immediately downstream of the capsid gene, as confirmed by whole genome sequencing. The genetic insertion served to increase the genome size of the wild type T7Select by less than 3% (37.3 kb vs. 38.3 kb) leaving it with a genome still smaller (~38 kb) than that of its 39.7 kb wild-type counterpart [54].

During infection, T7 DNA is replicated in concatemers and successful phage maturation relies on the specific recognition and cleavage of cos sites located at the termini of each genomic copy. If the genetic insertion is too large, then progeny phages will be unable to fit the genome into the finite space provided within the capsid.

**Figure 3:**
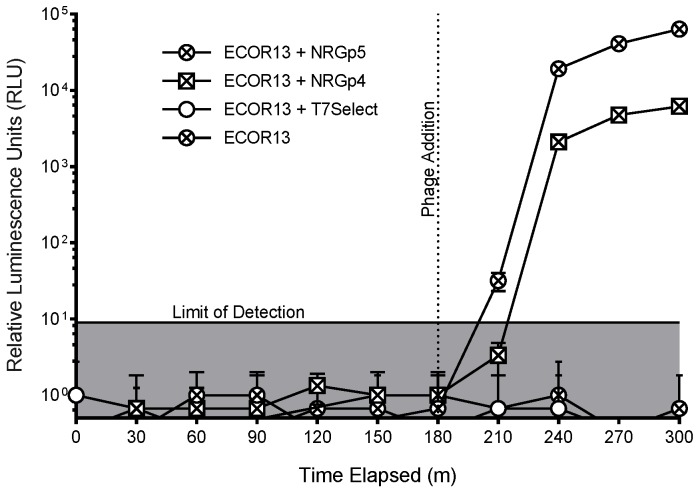
Luminescence was measured from *E. coli* samples with and without the phages of T7 Select, which did not contain a gene for a reporter enzyme, NRGp4, but which contained a gene for NanoLuc-CBM, and NRGp5, which contained an optimized NanoLuc-CBM gene. It can be seen that the reporter genes resulted in luminescence with the optimized gene providing a higher signal. Error bars indicate the standard deviation of three biological triplicates and the limit of detection (lowest positive signal) was calculated as the negative control + 3x the standard deviation. The standard deviation for phages NRGp4 and NRGp5 was too low to produce visible error bars at the final time points of the assay.

Packing of the modified genome into the capsid was not expected to cause a loss in fitness as larger reporter genes have previously been inserted into T7 with no apparent lack of fitness [55,56]. As seen in Figure 3, only cultures infected with phage NRGp4 or NRGp5 produced luminescent signals above the limit of detection. While changes to the promoter and ribosome binding sites upstream of the reporter only served to decrease the luminescence signal during the infection of identical cultures (data not shown), removal of the pelB N-terminal secretion signal used in phage NRGp4 [48] produced nearly a half log increase in signal intensity (Figure 3) when identical cultures were infected and compared. 

The results suggest that the newly developed reporter phage NRGp5 is a viable candidate for use as a more sensitive biosensor element in a syringe-based detection assay.

**Figure 4:**
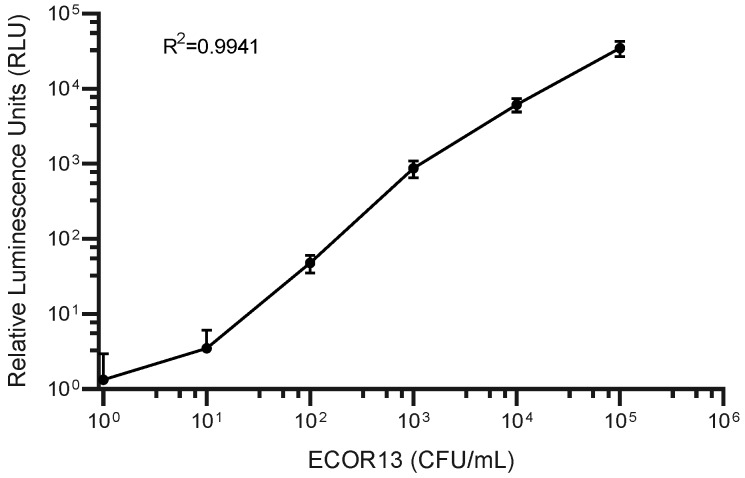
NRGp5 was added at a high multiplicity of infection (MOI) to varying concentrations of *E. coli* ECOR13. The luminescence of the lysate resulting from the infections was measured following the addition of substrate. The results suggest relative relationship (R^2^ = 0.9941) between the concentration of *E. coli* and the generation of the luminescent signal over several orders of magnitude.

**Figure 5:**
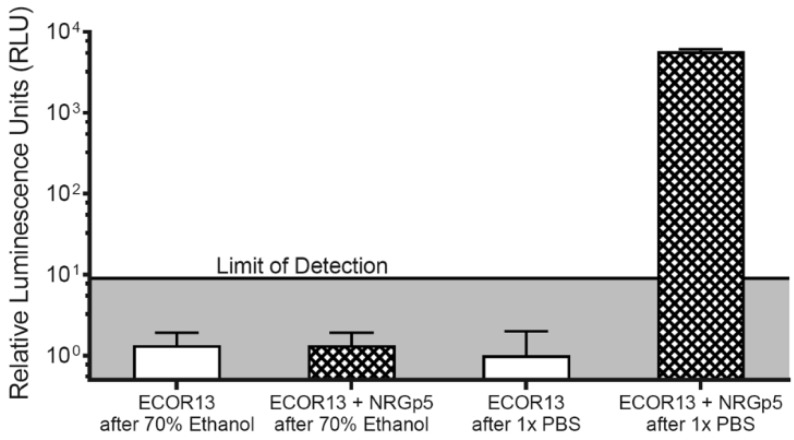
To demonstrate the ability of the phage-based assay to distinguish between viable and non-viable *E. coli* ECOR13 cells, 10^3^ bacterial cells were exposed to either 70% ethanol or PBS, and washed. The cells were then either infected with NRGp5 or incubated without phages. The only cell treatment regime to display luminescence following incubation and substrate addition was the non-ethanol treated *E. coli* ECOR13 infected with NRGp5.

In order to make an initial estimate of the potential to detect low concentrations of CFUs, NRGp5 was used to infect a known high concentration of *E. coli* ECOR13. The lysate was then serially diluted and tested (Figure 4). From this we can see that the lysate from an equivalent to low numbers of *E. coli* were detectable. As seen in Figure 4, the results suggested a relatively linear response (R^2^ = 0.9941) over a broad range of bacterial concentrations 10^0^–10^5^ CFU/mL. Furthermore, because drinking water often undergoes treatment steps in order to kill potentially harmful pathogens, the ability to distinguish between viable and non-viable bacterial is critical for reliable results. Given that phages utilize the genetic machinery of the host bacteria for successful replication, it was not expected that NRGp5 could replicate in non-viable cells. In order to determine if the assay is able to distinguish between viable and non-viable bacterial cells, *E. coli* ECOR13 cells were treated with 70% ethanol and washed prior to analysis. As demonstrated in Figure 5, only phages added to non-ethanol treated cells were able to produce luminescence following substrate addition, while bacterial cells treated with ethanol did not produce luminescence following phage addition.

### 3.3. Phage-Based Syringe Filter Detection Assay

**Figure 6:**
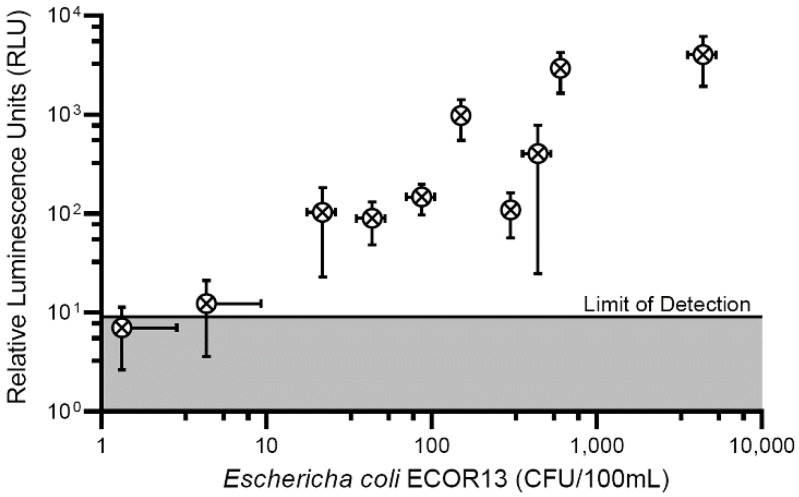
Performance of the phage-based syringe filter detection assay compared to standard plate counts. Water (100 mL) was processed through a syringe filter (0.22 um, regenerated cellulose) where bacteria (*E. coli* ECOR13) were enriched prior to the addition of phage NRGp5. Reporter enzymes were expressed, immobilized onto the cellulose filter, and luminescence was measured.

After determining the sensitivity of the phage to produce a measurable signal in bulk solution we introduced a sample concentration to further improve the performance of the phage-based assay. The addition of a syringe filter to the detection scheme served to decrease the limit of detection several orders of magnitude from ~100 CFU/mL to less than 20 CFU/100 mL (Figure 6). The inoculation levels used to determine the aforementioned detection limits were low enough to achieve the fractional recovery of positive results as recommended by multiple validation procedures [57,58]. As indicated by the horizontal error bars in Figure 6 (error bars crossing an axis are not displayed) recovery of positive samples at low inoculation levels was possible in only approximately 80% of the samples. 

In addition to fractional recovery of positive samples, other considerations towards methodology validation were considered to ensure the robustness of the newly developed detection assay. Recovery and enrichment steps were included prior to phage infection to provide injured and/or stressed bacterial cells an opportunity to recover. The test was performed using target organisms (*E. coli*) in the intended product (drinking water) where the organism has limited growth potential from the lack of available nutrients. Finally, our novel phage-based method was directly compared to a gold standard reference culture-based method to ensure accurate quantitation of bacterial inoculum.

Our novel detection assay realized multiple improvements over previously developed technologies and approaches the limits of detection of standard culture-based techniques in only 5 h, compared to 24–48 h for the latter methods. Phage-based detection schemes have the potential to realize further improvements to the limit of detection at these bacterial concentrations while maintaining a shorter assay time than standard reference methods.

## 4. Conclusions

The presented research displays a specific, rapid, and effective detection assay for indicators of *E. coli* in large drinking water samples based on heterologous enzyme expression via phage infection. Phages have multiple properties that make them excellent candidates as biorecognition elements for the sensitive and rapid detection of bacteria. Not only are phages incredibly species specific, they are also capable of differentiating between live and non-viable cells.

We have demonstrated the successful insertion of a reporter enzyme cassette into a phage genome to create the reporter phage NRGp5. This recombinant phage forced the overexpression of a highly active bifunctional reporter enzyme that was immobilized onto a regenerated cellulose membrane filter, which facilitated the rapid detection of low concentrations of indicator bacterial *E. coli* cells. While a cocktail of phages is typically needed to cover a broad host range, this phage-based platform offers portability and low cost with rapid results. Improvements in enzymatic activity and bacterial expression as well as phage host range will serve to create a robust and sensitive detection assay with potential to dramatically improve the lives of people around the world through the rapid microbiological analysis of vital drinking water supplies.
